# Supplementary figures and images for: Longitudinal map of transcriptome changes in the Lyme pathogen Borrelia burgdorferi during tick-borne transmission
Source: eLife. 2023 Jul 14;12:RP86636. doi: 10.7554/eLife.86636 (PMC10393048; doi:10.7554/eLife.86636)

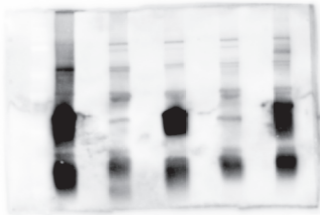

Supplement: Figure 1—figure supplement 1—source data 1. [file elife-86636-fig1-figsupp1-data1.pdf]

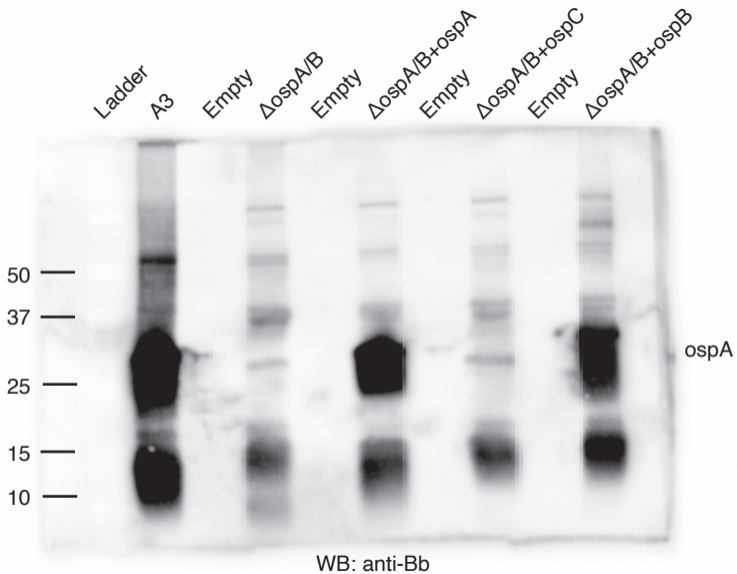

Supplement: Figure 1—figure supplement 1—source data 2. — OspB and OspC restored lanes are not included in the figure for simplicity. [file elife-86636-fig1-figsupp1-data2.pdf]
